# Effect of juggling expertise on pointing performance in peripheral vision

**DOI:** 10.1371/journal.pone.0306630

**Published:** 2024-07-12

**Authors:** Tristan Jurkiewicz, Ludovic Delporte, Patrice Revol, Yves Rossetti, Laure Pisella

**Affiliations:** 1 Centre de Recherche en Neurosciences de Lyon, Trajectoires Team, Bron, France; 2 Centre d’Exploration de la Rétine Kléber, Ophthalmology Department, Lyon, France; 3 Plateforme Mouvement et Handicap, Hôpital Henry Gabrielle, St-Genis-Laval, France; The Ohio State University, UNITED STATES OF AMERICA

## Abstract

Juggling is a very complex activity requiring motor, visual and coordination skills. Expert jugglers experience a “third eye” monitoring leftward and rightward ball zenith positions alternately, in the upper visual fields, while maintaining their gaze straight-ahead. This “third eye” reduces their motor noise (improved body stability and decrease in hand movement variability) as it avoids the numerous head and eye movements that add noise into the system and make trajectories more uncertain. Neuroimaging studies have shown that learning to juggle induces white and grey matter hypertrophy at the posterior intraparietal sulcus. Damage to this brain region leads to optic ataxia, a clinical condition characterised by peripheral pointing bias toward gaze position. We predicted that expert jugglers would, conversely, present better accuracy in a peripheral pointing task. The mean pointing accuracy of expert jugglers was better for peripheral pointing within the upper visual field, compatible with their subjective experience of the “third eye”. Further analyses showed that experts exhibited much less between-subject variability than beginners, reinforcing the interpretation of a vertically asymmetrical calibration of peripheral space, characteristic of juggling and homogenous in the expert group. On the contrary, individual pointing variability did not differ between groups neither globally nor in any sector of space, showing that the reduced motor noise of experts in juggling did not transfer to pointing. It is concluded that the plasticity of the posterior intraparietal sulcus related to juggling expertise does not consist of globally improved visual-to-motor ability. It rather consists of peripheral space calibration by practicing horizontal covert shifts of the attentional spotlight within the upper visual field, between left and right ball zenith positions.

## Introduction

**Fig 1:**
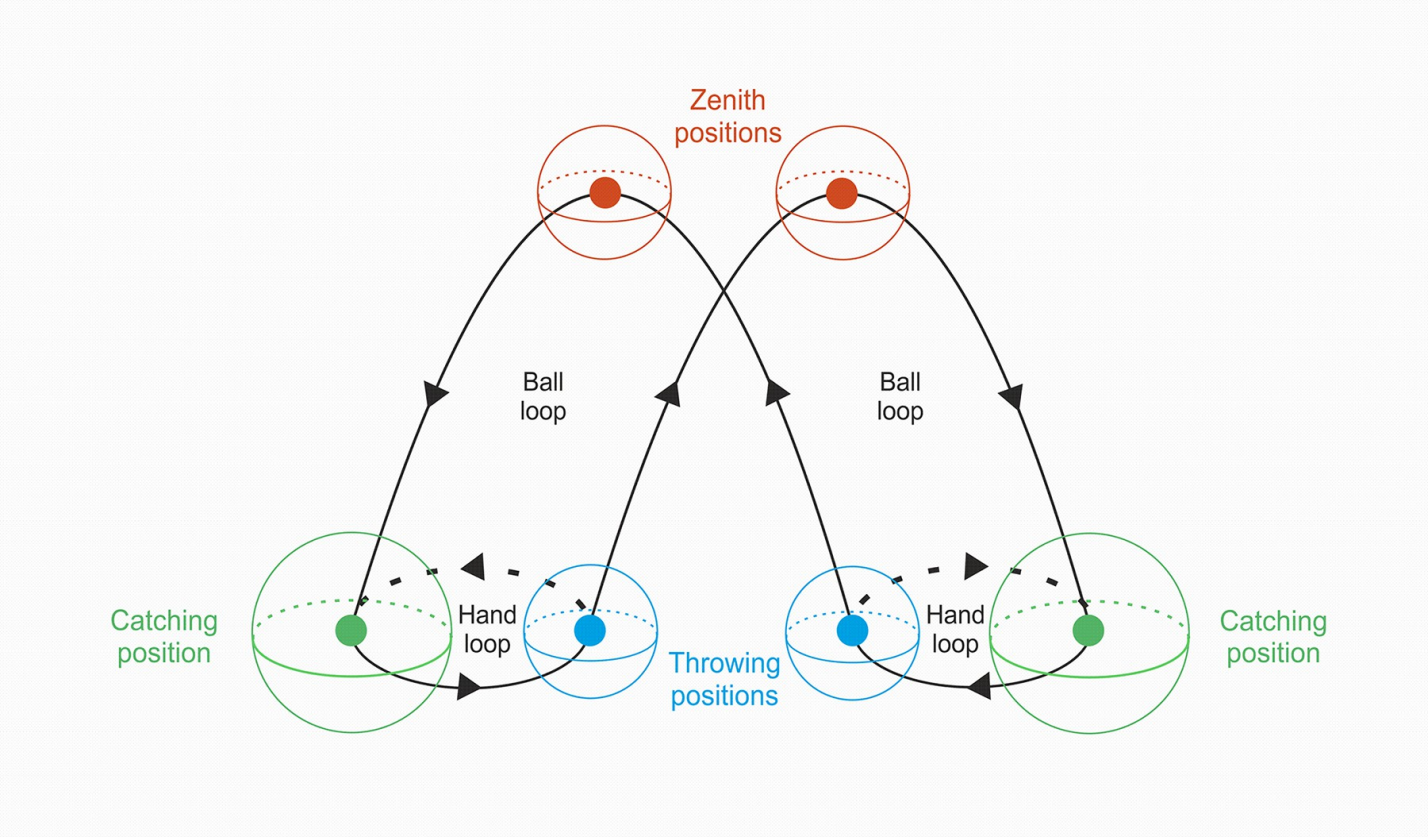
Cascade juggling scheme (adapted from Huys and Beek 2002 [3]). The blue dots correspond to the ball’s average throwing positions, the green dots to the ball’s average catching positions and the red dots to the ball’s average zenith positions. The comparative volumes of the spheres are representative of the maximal variation of ball positions retained to categorize expert jugglers (see Method).

Juggling is a very complex activity requiring motor, visual, tactile, kinaesthetic, postural, as well as motion perception and between arm coordination skills. Visually, it implies being able to monitor the trajectory of several balls simultaneously which requires covert attention in peripheral vision. In line with the subjective jugglers’ experience of a “third eye”, a constant position is fixated ahead but the attentional spotlight monitors the upper visual fields, leftward and rightward alternately, to determine when the ball is at zenith position (Fig 1) to throw the next ball. This would avoid the numerous head and eye movements of beginning jugglers that make trajectories more uncertain [1, 2].

Learning to juggle is accompanied by plastic changes in brain structure. During the learning, plasticity has been evidenced as a gain in the grey matter volume in the visual area V5/MT, which is the perceptive area of motion (medial temporal area), in a majority of studies involving either whole-brain analysis or specific focus on this region of interest [4–8]. Two of these studies [6, 8] have also assessed specifically and highlighted a grey matter volume gain in the posterior part of the intraparietal sulcus (IPS), which pertains to the network of the posterior parietal cortex (PPC) specific for reaching to peripheral targets [9]. An increase of the fractional anisotropy in the white matter under the posterior IPS has also been observed [10]. These studies suggest that learning to juggle strengthens the white and grey matter of the dorsal visual stream, which conducts visual information from occipital to PPC for action [11, 12] and is also involved in the perception of peripheral visual space [13, 14]. Our hypothesis is that juggling expertise improves peripheral vision and therefore a good juggler would perform better than a beginner in other activities that involve peripheral vision.

The posterior parietal network specific for reaching to peripheral targets [9], and more precisely the posterior IPS at the parieto-occipital junction, has been identified as the critical lesion site of optic ataxia [15], a neurological condition characterized by imprecise reaching movements in peripheral vision [16–19]. Patients’ pointing errors in the contralesional visual field consist of hypometria toward gaze position, increasing with visual target eccentricity [17, 18, 20]. A modeling of these hypometric errors revealed a logarithmic underestimation of visual target eccentricities similar to the equation modeling the central vision magnification and the compression of peripheral space characteristic of most subcortical and cortical visual areas [18]. Central vision is indeed over-represented in the superior colliculus, the primary visual area and the visual areas of the ventral visual stream at the expense of peripheral vision [21–25]. In contrast, peripheral vision is fairly represented in the dorsal visual stream [26], as if one of its functional roles was to actively compensate for the under-representation of peripheral vision [18] for accurate perceptual metrics (« Where ») and interaction with environmental space (« How »). This functional role would be evidenced by optic ataxia deficits [18] caused by posterior IPS damage, or by its hypertrophy reflecting the intensive practice of peripheral vision as investigated in the present paper for juggling. To test whether juggling improves peripheral vision, especially in the upper visual field in line with the subjective experience of jugglers’ third eye, we compared peripheral pointing performance between beginner and expert jugglers. Such behavioural difference would fit the reported hypertrophy of the posterior IPS in expert jugglers [8]). The secondary question deals with whether such pointing performance improvement would be related to better motor ability or to better perceptual localisation of the peripheral visual targets.

## Material and method

### Experimental design

To test our hypothesis, we recruited expert (mostly professionals or non-professional advanced jugglers with decades of regular practice) and beginner jugglers for a prospective monocentric study (between March 22, 2019 and January 31, 2020). Sixteen subjects volunteered for this experiment which was run at the Movement and Handicap motion analysis facilities (https://www.chu-lyon.fr/plateforme-mouvement-et-handicap). Mean participants age was 28.7 ± 6.9 years (ranging from 20 to 40 years). For this study, written consent was obtained from each participant in accordance with the Ethics Evaluation Committee of Inserm (EECI) 2019 approval n° 19–569.

Subjects started with a pointing task toward targets presented in peripheral vision and then a juggling task. Movements were recorded using an optoelectronic system (3D Motion Analysis®). This device was composed of 7 cameras with infrared emitters connected to a central processing unit. This system allowed recording the three-dimensional displacement of passive sensors stuck on the subject or on objects in a space. The sensors were spheres of 5 to 14 mm in diameter covered with an adhesive called "scotch light". This adhesive reflected the infrared emitted by the optoelectronic cameras (passive sensors). For the pointing task, subjects had a 5 mm diameter sensor stuck on their right index finger to record the hand movement. For the juggling task, passive sensors were stuck on subjects’ shoulders and elbows, and the juggling balls were entirely covered with reflective tape to record their trajectories.

During juggling, the position of the individual’s center of gravity was recorded in order to determine the effect of juggling on the subject’s posture. A 6-axis force platform of dimensions 60*40cm was used. It was used to record the forces (N) and moments (N*m) applied to it. This made it possible to calculate the instantaneous projection of the person’s center of gravity on the platform, and to track its displacements.

**Fig 2:**
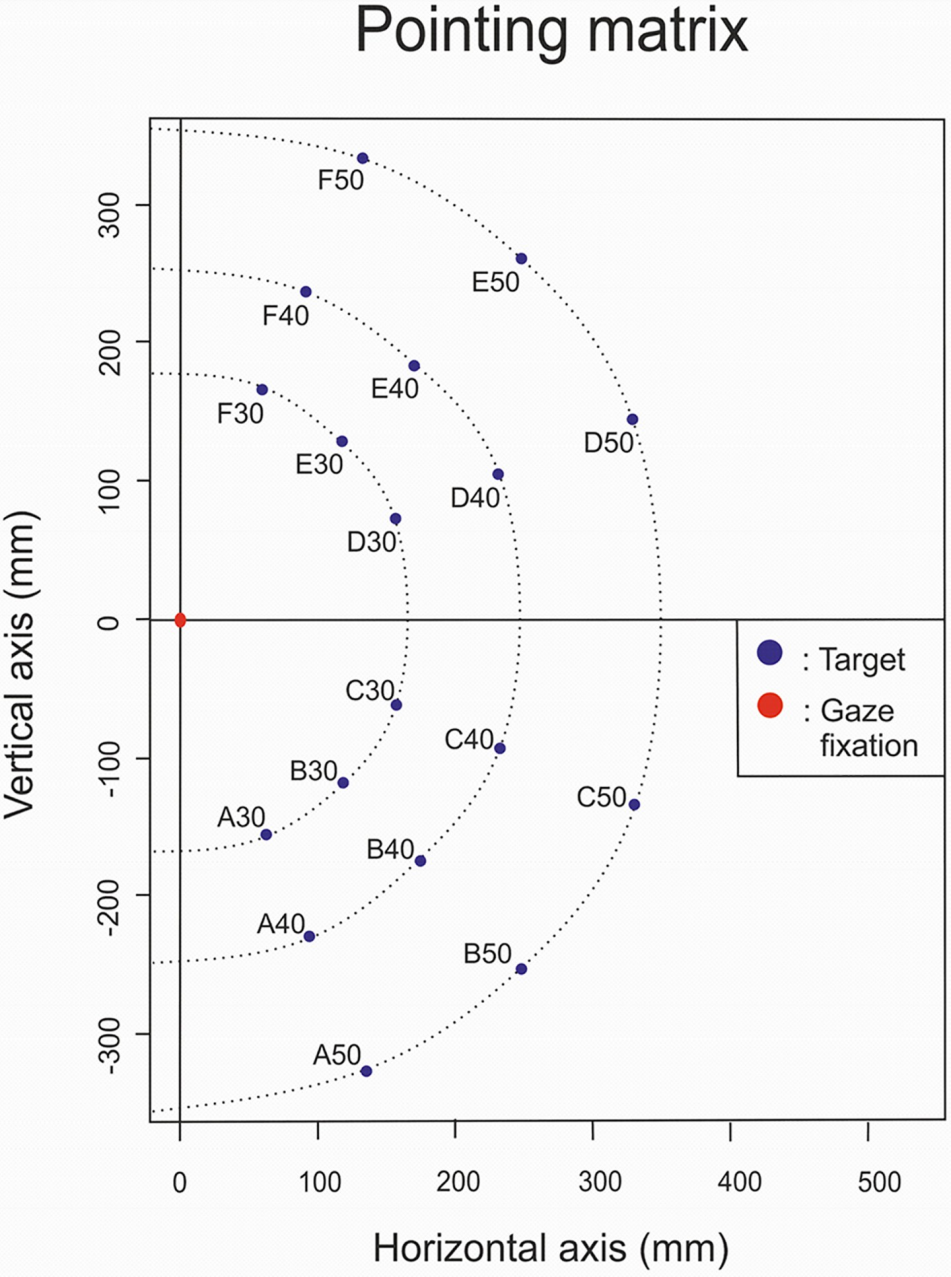
Pointing task matrix: targets were presented at 30°, 40° and 50° of visual eccentricities relative to central ocular fixation dot (red dot) and along three directional axes in the lower visual field (A, B and C) and in the upper visual field (D, E et F). Directional axes corresponded to angles of -67.5° (Axis A), -45° (Axis B), -22.5° (Axis C), +22.5° (Axis D), +45° (Axis E) and +67.5° (Axis F) relative to the horizontal axis comprising the ocular fixation dot.

For the pointing task, subjects were placed 30 cm in front of a vertical frontal pointing screen on which visual targets were randomly presented with a laser device. When both the laser was activated by the examiner and the patient pressed the start button, the light of the target was projected on the pointing screen. As soon as the patient released the button to point toward the peripheral visual stimulus, the target disappeared. The position of the eyes was monitored with a custom electro-oculogram to ensure that participants maintain central ocular fixation and that the targets were presented at the correct eccentricity. Three visual eccentricities were tested on six axes in the upper and the lower visual field (for a total of eighteen targets locations) (Fig 2). Each target was tested at least 6 times (108 trials per subject). As soon as one target was not perceived by participant (missing data), a 7th repetition was performed (126 trials). Missing data corresponded to an average of 1.55 ± 1.42% of trials for the experts (between 0 and 3.17%; i.e. 4/126 trials for the maximum) and to an average of 2.56 ± 2.44% of trials (between 0 and 7.14%; i.e. a maximum of 7/126 trials) for beginners. The final pointing positions were extracted from the Motion Analysis 3D® recording and compared with the target position (pointed in free viewing condition at the end by each subject) to obtain pointing errors in mm in the screen plane (2D).

### Participants

Inclusion criteria were to be able to juggle in a cascade with three balls for at least 15 seconds. The only exclusion criterion was the presence of a motor or visual impairment that could influence juggling.

To be considered as an expert, subjects had to meet a majority of the following five expertise criteria:

- Elbow/shoulder amplitude ratio >1,5; with expertise, there is a decrease in the maximum angle reached by the shoulder and an increase in the angle reached by the elbow [27].
- Area of the center of pressure during the juggling task <40cm^2^; with expertise, improved body stability and decrease in hand movement variability is observed [2, 28, 29].
- Volume of the position of the balls <1,5dm^3^ at zenith, <1,5dm^3^ at throwing and <3,75dm^3^ at catching positions); with expertise, there is a reduced variability of ball positions [27]. The volume considered for the catching position is 2.5 times more important than those of zenith and throwing positions (Fig 1), whatever the level of expertise.

Following this analysis, nine participants were assigned to the Beginner group and seven to the Expert juggler group (Table 1).

**Table 1:** Juggling parameter averages by group.

		Volume at catching position (dm^3^)	Volume at zenith position (dm^3^)	Volume at throwing position (dm^3^)	Area of the center of pressure (cm^2^)	Elbow/shoulder amplitude ratio
Experts	Mean	4,09	1,48	1,04	25,14	2,85
	SD	2,68	0,68	0,87	14,36	1,62
Beginners	Mean	32,75	10,43	5,89	87,75	1,47
	SD	51,74	20,43	4,99	103,76	0,79

### Statistical analysis of peripheral pointing performance

**Fig 3:**
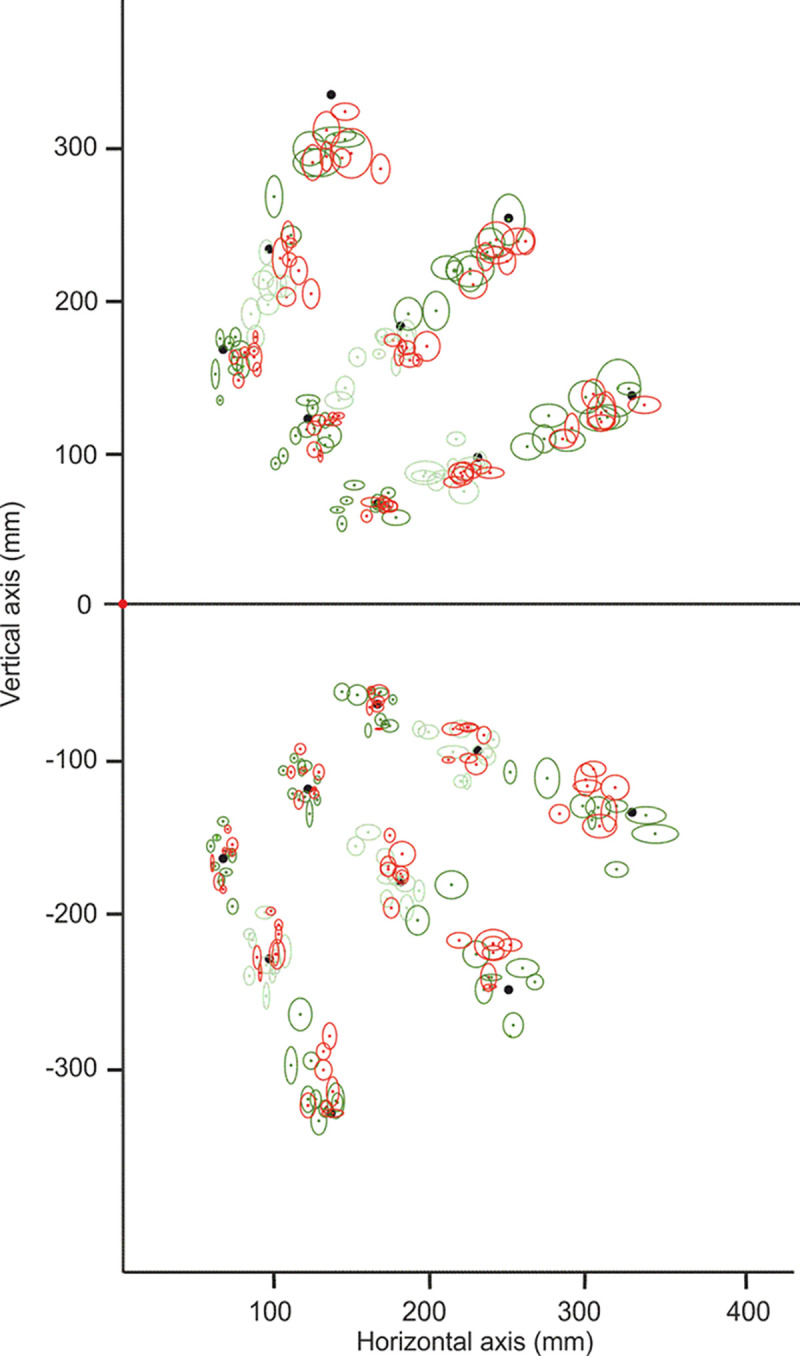
Individual pointing performance on each target of the matrix—Illustration of intra-individual pointing variability (confidence ellipses surface) and averaged pointing error (center of ellipses to be compared to target positions materialised by black dots) for beginners (in green) and experts (in red) jugglers. Darker colours were used for endpoints corresponding to the less and the most eccentric targets. Confidence ellipse axes are standard deviations along X and Y dimensions for each individual and each target of the matrix.

We used Statistica V14.0.0.15 software for Windows (StatSoft, Inc.). Descriptive statistics (mean and standard deviation) of participants’ pointing errors were computed along X and Y dimensions separately, for each of the three visual target eccentricities and each of the three axes of the lower and upper visual fields (Fig 3). Pointing errors were signed negative in case of a bias toward the gaze fixation point, i.e. gaze-centred hypometria.

We then ran repeated measure ANOVAs on individual pointing error mean and standard deviation (SD), reflecting pointing accuracy and intra-individual variability, respectively, in X and Y dimensions, with Visual field (lower vs upper), Eccentricity (30°, 40°, 50°) and Group (beginners vs experts) as main factors.

We also performed ANOVA with targets of the matrix as repeated measures testing the effect of Visual field and Group on the within-group variability of mean pointing accuracy.

## Results

### Intra-individual pointing variability across groups, visual fields and eccentricities

Since experts have been selected based on their smaller motor noise in juggling, attested by their elbow/shoulder amplitude ratio, their smaller area of center of pressure, and their smaller volume of ball positions (Table 1), we first assessed whether they also exhibited smaller intra-individual variability than beginners in (untrained) peripheral pointing task. Repeated measure ANOVA Group comparison (beginners/experts) of intra-individual endpoints variability (SD) along the X dimension with Visual field (upper/lower) and Eccentricities (30°, 40° and 50°) factors showed a main effect of Eccentricity (F(2,28) = 157.7, p<0.01, η^2^ = 0.92) and a significant interaction between Visual field and Eccentricity (F(2,28) = 5.14, p<0.05, η^2^ = 0.27). Importantly, there were no main effect of Group (F(1,14) = 0.7, p = 0.43, η^2^ = 0.047) and no significant interaction involving the group (all p>0.05, all F< 0.7, all η^2^<0.048). This means that, similarly in the two groups, the pointing standard deviation increases more with target eccentricity in the upper visual field (Fig 3).

A similar ANOVA was computed for the Y dimension. We found the same main effect of Eccentricity (F(2, 28) = 58.28, p<0.01, η^2^ = 0.81), endpoint variability increasing when target eccentricity increases. We also found a main effect of Visual field (F(1, 14) = 30.99, p<0.01, η^2^ = 0.69) with higher variability in the upper visual field. There was no interaction between Visual field and Eccentricity (F(2, 28) = 2,31, p = 0.12, η^2^ = 0.14). Importantly again, there was no main effect of Group (F(1, 14) = 1,52, p = 0.24, η^2^ = 0.098) and no significant interaction involving the group (all p>0.05, all F<4.32, all η^2^ = 0.24). This means that, in spite of their smaller motor noise in juggling (Table 1), expert individuals did not exhibit smaller pointing variability.

### Averaged pointing error across groups, visual fields and eccentricities

Pointing errors along X and Y dimensions were most often negative, revealing an overall pointing bias toward the gaze fixation point (Fig 3).

Repeated measure ANOVA Group comparison (beginners/experts) of individual endpoints means with Visual field (upper/lower) and Eccentricities (30°, 40° and 50°) factors showed a significant main effect of Eccentricity along X (F(2,28) = 68.57, p<0.001, η^2^ = 0.83) and Y (F(2,28) = 27.80, p<0.001, η^2^ = 0.67) dimensions; which means that, in both groups, the gaze-centred hypometria increased when the eccentricity of targets increased.

There was also a significant interaction between Visual field and Group (F(1,14) = 6.42, p = 0.024, η^2^ = 0.31) along the X dimension (this was not the case along the Y dimension: F(1,14) = 1.27, p = 0.28, η^2^ = 0.083), reflecting that the horizontal pointing bias was smaller for the experts compared to the beginners jugglers but only within the upper visual field.

### Comparison of inter-individual variability of pointing accuracy across groups and visual fields

**Fig 4:**
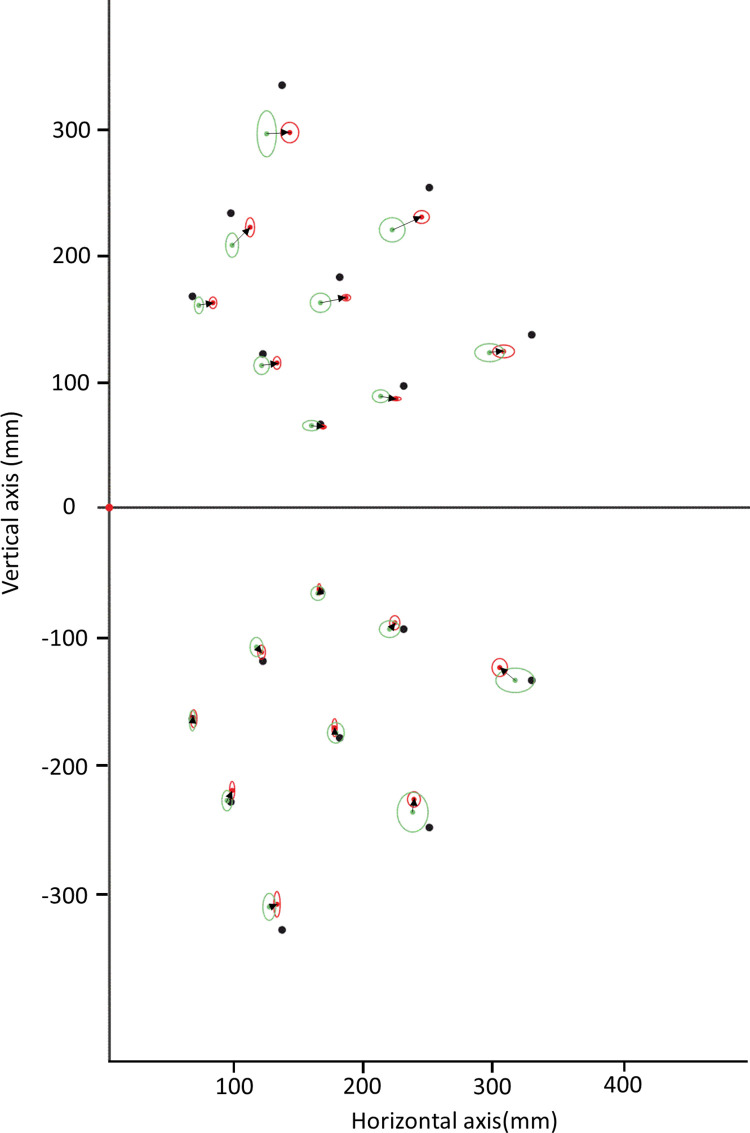
Mean pointing error per target and group (green dot for beginners and red dot for experts) and inter-individual variability (green ellipse for beginners and red ellipse for experts). The black arrows drawn from the mean endpoints of beginners to those of experts show a pointing accuracy gain toward the targets (black dots) for juggling experts in the upper visual field.

In order to assess whether the better pointing accuracy of the expert group in the upper visual field, specific to the horizontal dimension, was a homogenous characteristic among the experts, we compared the inter-individual behavioural homogeneity within groups. This analysis revealed that experts exhibited much less between-subject variability than beginners (see Fig 4). To evaluate this effect, the different targets were used to run a repeated measure ANOVAs on the inter-individual standard deviation along the X and the Y dimensions, with Group (beginners/experts) and Visual field (upper/lower) as factors. We found a significant main effect of Group along the X dimension (F(1, 16) = 34.27, p<0.001, η^2^ = 0.68) and the Y dimension (F(1, 16) = 21.04, p<0.001, η^2^ = 0.57). There was no main effect of Visual field (X dimension: F(1, 16) = 0.43, p = 0.52, η^2^ = 0.026 and Y dimension F(1, 16) = 1.20, p = 0.29, η^2^ = 0.070) and no interaction (X dimension: F(1, 16) = 1,10, p = 0.31, η^2^ = 0.064 and Y dimension F(1, 16) = 1.33, p = 0.27, η^2^ = 0.077).

This substantial decrease of inter-individual standard deviation among the experts compared to the beginners suggested that the individuals of the experts group had a more homogenous peripheral pointing accuracy across visual fields.

## Discussion

Neuroimaging studies have shown that learning to juggle induces white and grey matter hypertrophy at the posterior IPS [6–8, 10]. Damage to the posterior IPS leads to optic ataxia (OA), a clinical condition typically affecting reaching in peripheral vision while fixating straight-ahead [15, 17, 18, 30, 31]. More precisely, OA patients display an increase in mean accuracy bias in the contralesional visual field with more pointing variability when they use their contralesional hand [17, 18]. Increased motor variability is frequent in clinical conditions for movements made by the contralesional limb irrespective of lesion localization. It is therefore assumed that this parameter reflects a general motor noise non-specific to OA. In contrast, gaze-related peripheral pointing bias appeared specific to posterior IPS lesion and optic ataxia [17, 18, 31]. We, therefore, predicted that expert jugglers would present a smaller pointing bias, i.e. a better accuracy in a peripheral pointing task, specifically in the upper visual field. As a matter of facts, this localization corresponds to their trained experience of tracking the balls’ trajectory zenith positions while fixating straight-ahead.

Our experiment yielded three main results. First, the individual pointing variability did not differ between experts and beginners, neither globally nor in any sector of space. Second, and in contrast, the mean pointing accuracy was better for expert jugglers for peripheral pointing within the upper visual field (Fig 3). Third, further analyses showed that individuals of the expert group also point more homogeneously than beginners: inter-individual variability of pointing accuracy was smaller across both the lower and upper visual fields in the expert group. Altogether, these results support the idea of a specific pointing pattern in expert jugglers whose characteristic is to be asymmetrical with a better accuracy in the upper visual field than in the lower. Our interpretation of this vertically-asymmetrical pattern is that their ability of positional encoding in peripheral space has been calibrated by practicing the “third eye” monitoring of balls zenith positions during juggling.

The posterior IPS pertains to the dorsal visual stream, whose functional role in spatial representation/attention (« Where »: spatial perception) versus action programming/intention (« How »: visual-to-motor transformation) remains debated [13, 32–36]. We can speculate that if, according to Milner & Goodale [11, 12] theoretical dual-stream model, the dorsal visual stream is dedicated to action, then the posterior IPS-based juggling expertise would correspond to their smaller motor noise in juggling (attested by the elbow/shoulder amplitude ratio, the smaller area of center of pressure, and the smaller volume of ball positions) that would have transferred to pointing. In contrast, experts exhibited pointing variability similar to beginners. Alternatively, the dorsal visual stream has been more recently proposed to crucially improve pointing accuracy in peripheral vision via a representation of space that compensates for its under-representation in other visual areas [18]. Such improvement of spatial resolution in visual periphery is observed with covert attention [37], also relying on the IPS (dorsal attentional network [38]), and could be beneficial for both for vision-for-action and vision-for-perception. In line with this interpretation of the dorsal visual stream dedicated to spatial processing, posterior IPS-based juggling expertise would correspond to a better accuracy of perceptual localization [39] at peripheral positions monitored in the upper visual field while juggling. Accordingly, the present study suggested that peripheral space processing is calibrated by juggling expertise, as attested by its asymmetry in favor of the upper visual field that is transferred to (untrained) pointing task.

Spatial accuracy in peripheral vision, both in motor or perceptual contexts, may depend on the ability to covertly shift attention to the target area. Indeed, the IPS lesion is responsible for reaching impairment in the contralesional visual field as well as for impaired detection [30, 40, 41] and discrimination [42, 43] of contralesional visual targets in covert orienting tasks. The homogenous improvement of the mean pointing performance of expert jugglers along the horizontal dimension could result from their trained capacity to shift attention laterally to balls’ left and right zenith positions. Adding the expertise argument to the former lesion argument, we therefore conclude that the present study adds evidence for the involvement of the dorsal visual stream in visuo-spatial encoding [19, 35, 39, 44–47], via its role in spatial attention [48, 49].

## Supporting information

S1 File (XLSX)

## References

[1] Dessing JC, Rey FP, Beek PJ. Gaze fixation improves the stability of expert juggling. Exp Brain Res. 2012;216:635–644. doi: 10.1007/s00221-011-2967-6 22143871 PMC3268979

[2] Rodrigues ST, Polastri PF, Gotardi GC, Aguiar SA, Mesaros MR, Pestana MB, et al. Postural Control During Cascade Ball Juggling: Effects of Expertise and Base of Support. Percept Mot Skills. 2016;123:279–294. doi: 10.1177/0031512516660718 27502243

[3] Huys R, Beek PJ. The coupling between point-of-gaze and ball movements in three-ball cascade juggling: the effects of expertise, pattern and tempo. J Sports Sci. 2002;20:171–186. doi: 10.1080/026404102317284745 11999474

[4] Boyke J, Driemeyer J, Gaser C, Büchel C, May A. Training-Induced Brain Structure Changes in the Elderly. J. Neurosci. 2008;28:7031–7035. doi: 10.1523/JNEUROSCI.0742-08.2008 18614670 PMC6670504

[5] Carius D, Andrä C, Clauß M, Ragert P, Bunk M, Mehnert J. Hemodynamic Response Alteration As a Function of Task Complexity and Expertise-An FNIRS Study in Jugglers. Front. Hum. Neurosci. 2016;10:126. doi: 10.3389/fnhum.2016.00126 27064925 PMC4811870

[6] Draganski B, Gaser C, Busch V, Schuierer G; Bogdahn U, May A. Neuroplasticity: Changes in Grey Matter Induced by Training. Nature. 2004;427:311–312. doi: 10.1038/427311a 14737157

[7] Driemeyer J, Boyke J, Gaser C, Büchel C, May A. Changes in gray matter induced by learning—revisited. PLoS One. 2008;23;3:e2669. doi: 10.1371/journal.pone.0002669 18648501 PMC2447176

[8] Gerber P, Schlaffke L, Heba S, Greenlee MW, Schultz T, Schmidt-Wilcke T. Juggling revisited—a voxel-based morphometry study with expert jugglers. Neuroimage. 2014;15:320–325. doi: 10.1016/j.neuroimage.2014.04.023 24736178

[9] Martin JA, Karnath HO, Himmelbach M. Revisiting the cortical system for peripheral reaching at the parieto-occipital junction. Cortex. 2015;64:363–379. doi: 10.1016/j.cortex.2014.11.012 25614234

[10] Scholz J, Klein MC, Behrens TE, Johansen-Berg H. Training induces changes in white-matter architecture. Nat Neurosci. 2009;12:1370–1371. doi: 10.1038/nn.2412 19820707 PMC2770457

[11] Milner AD, Goodale MA. The visual brain in action. 2nd ed. Oxford University Press; 2006.

[12] Milner AD, Goodale MA. Two visual systems re-viewed. Neuropsychologia. 2008;12;46:774–785. doi: 10.1016/j.neuropsychologia.2007.10.005 18037456

[13] Pisella L, Sergio L, Blangero A, Torchin H, Vighetto A, Rossetti Y. Optic ataxia and the function of the dorsal stream: contributions to perception and action. Neuropsychologia. 2009;47:3033–3044. doi: 10.1016/j.neuropsychologia.2009.06.020 19563817

[14] Jurkiewicz T, Salemme R, Froment C, Pisella L. Role of the Dorsal Posterior Parietal Cortex in the Accurate Perception of Object Magnitude in Peripheral Vision. Iperception. 2021;6;12:20416695211058476. doi: 10.1177/20416695211058476 34900214 PMC8652191

[15] Karnath HO, Perenin MT. Cortical control of visually guided reaching: evidence from patients with optic ataxia. Cereb Cortex. 2005;15:1561–1569. doi: 10.1093/cercor/bhi034 15716470

[16] Perenin MT, Vighetto A. Optic ataxia: a specific disruption in visuomotor mechanisms. I. Different aspects of the deficit in reaching for objects. Brain. 1988;111:643–674. doi: 10.1093/brain/111.3.643 3382915

[17] Blangero A, Ota H, Rossetti Y, Fujii T, Ohtake H, Tabuchi M, et al. Systematic retinotopic reaching error vectors in unilateral optic ataxia. Cortex. 2010;46:77–93. doi: 10.1016/j.cortex.2009.02.015 19345345

[18] Vindras P, Blangero A, Ota H, Reilly KT, Rossetti Y, Pisella L. The Pointing Errors in Optic Ataxia Reveal the Role of "Peripheral Magnification" of the PPC. Front Integr Neurosci. 2016;26;10:27. doi: 10.3389/fnint.2016.00027 27507938 PMC4960242

[19] Rossetti Y, Pisella L. Optic ataxia: beyond the dorsal stream cliché. In The Parietal lobes. Neurological and neuropsychological deficits A Volume in Handbook of Clinical Neurology; 2018. pp. 225–247. 10.1016/B978-0-444-63622-5.00011-5 29519460

[20] Pisella L, Rossetti Y, Rode G. Optic ataxia in Bálint-Holmes syndrome. Ann Phys Rehabil Med. 2017;60:148–154. 26874578 10.1016/j.rehab.2016.01.003

[21] Schwartz EL. Computational anatomy and functional architecture of striate cortex: a spatial mapping approach to perceptual coding. Vision Res. 1980;20:645–669. doi: 10.1016/0042-6989(80)90090-5 7445436

[22] Wade AR, Brewer AA, Rieger JW, Wandell BA. Functional measurements of human ventral occipital cortex: retinotopy and colour. Philos Trans R Soc Lond B Biol Sci. 2002;29:963–973. doi: 10.1098/rstb.2002.1108 12217168 PMC1693014

[23] Polimeni JR, Balasubramanian M, Schwartz EL. Multi-area visuotopic map complexes in macaque striate and extra-striate cortex. Vision Res. 2006;46:3336–3359. doi: 10.1016/j.visres.2006.03.006 16831455 PMC2248457

[24] Schira MM, Wade AR, Tyler CW. Two-dimensional mapping of the central and parafoveal visual field to human visual cortex. J Neurophysiol. 2007;97:4284–4295. doi: 10.1152/jn.00972.2006 17360817

[25] Wu J, Yan T, Zhang Z, Jin F, Guo Q. Retinotopic mapping of the peripheral visual field to human visual cortex by functional magnetic resonance imaging. Hum Brain Mapp. 2012;33:1727–1740. doi: 10.1002/hbm.21324 22438122 PMC6870278

[26] Pitzalis S, Fattori P, Galletti C. The human cortical areas V6 and V6A. Vis Neurosci. 2015;32:E007. doi: 10.1017/S0952523815000048 26241369

[27] Hashizume K, Matsuo T. Temporal and spatial factors reflecting performance improvement during learning three-ball cascade juggling. Hum Mov Sci. 2004;23:207–233. doi: 10.1016/j.humov.2004.08.003 15474178

[28] Leroy D, Thouvarecq R, Gautier G. Postural organisation during cascade juggling: influence of expertise. Gait Posture. 2008;28:265–270. doi: 10.1016/j.gaitpost.2007.12.071 18262422

[29] Mapelli A, Galante D, Paganoni S, Fusini L, Forlani G, Sforza C. Three-dimensional hand movements during the execution of ball juggling: effect of expertise in street performers. J Electromyogr Kinesiol. 2012;22:859–865. doi: 10.1016/j.jelekin.2012.05.010 22763234

[30] Rossetti Y, Revol P, McIntosh R, Pisella L, Rode G, Danckert J, et al. Visually guided reaching: bilateral posterior parietal lesions cause a switch from fast visuomotor to slow cognitive control. Neuropsychologia. 2005;43(2):162–77. doi: 10.1016/j.neuropsychologia.2004.11.004 15707902

[31] Dijkerman H C, McIntosh R D, Anema H A, de Haan E H F, Kappelle L J, Milner A D. Reaching errors in optic ataxia are linked to eye position rather than head or body position Neuropsychologia 2006;44(13):2766–73. 16321407 10.1016/j.neuropsychologia.2005.10.018

[32] Colby CL, Goldberg ME. Space and attention in parietal cortex. Annu Rev Neurosci. 1999;22:319–349. doi: 10.1146/annurev.neuro.22.1.319 10202542

[33] Andersen RA, Buneo CA. Intentional maps in posterior parietal cortex. Annu Rev Neurosci. 2002;25:189–220. Andersen, R. A. and Buneo, C. A. (2002) Intentional maps in posterior parietal cortex. Annual Review of Neuroscience, 25, 189–220 doi: 10.1146/annurev.neuro.25.112701.142922 12052908

[34] Hwang EJ, Hauschild M, Wilke M, Andersen RA. Inactivation of the parietal reach region causes optic ataxia, impairing reaches but not saccades. Neuron. 2012;6;76:1021–1029. 10.1016/j.neuron.2012.10.030 PMC3597097 23217749

[35] Cavina-Pratesi C, Connolly JD, Milner AD. Optic ataxia as a model to investigate the role of the posterior parietal cortex in visually guided action: evidence from studies of patient M.H. Front Hum Neurosci. 2013; 16;7:336. 10.3389/fnhum.2013.00336 PMC3712225 23882200

[36] Andersen RA, Andersen KN, Hwang EJ, Hauschild M. Optic ataxia: from Balint’s syndrome to the parietal reach region. Neuron. 2014; 5:81:967–983; 10.1016/j.neuron.2014.02.025 PMC4000741 24607223

[37] Yeshurun Y, Carrasco M. Attention improves or impairs visual performance by enhancing spatial resolution. Nature. 1998;396:72–75. doi: 10.1038/23936 9817201 PMC3825508

[38] Corbetta M. Frontoparietal cortical networks for directing attention and the eye to visual locations: identical, independent, or overlapping neural systems? Proc Natl Acad Sci U S A. 1998; 3;95:831–838. doi: 10.1073/pnas.95.3.831 9448248 PMC33805

[39] Vialatte A, Yeshurun Y, Khan AZ, Rosenholtz R, Pisella L. Superior Parietal Lobule: A Role in Relative Localization of Multiple Different Elements. Cereb Cortex. 2021. 31(1):658–671. doi: 10.1093/cercor/bhaa250 32959044

[40] Striemer C, Blangero A, Rossetti Y, Boisson D, Rode G, Vighetto A, et al. Deficits in peripheral visual attention in patients with optic ataxia. Neuroreport. 2007;18:1171–1175. doi: 10.1097/WNR.0b013e32820049bd 17589321

[41] Gillebert CR, Mantini D, Thijs V, Sunaert S, Dupont P, Vandenberghe R. Lesion evidence for the critical role of the intraparietal sulcus in spatial attention. Brain. 2011;134:1694–1709. doi: 10.1093/brain/awr085 21576110

[42] Blangero A, Khan AZ, Salemme R, Deubel H, Schneider WX, Rode G, et al. Pre-saccadic perceptual facilitation can occur without covert orienting of attention. Cortex. 2010;46:1132–1137. doi: 10.1016/j.cortex.2009.06.014 19660745

[43] Martin-Arevalo E, Guedj C, Cotton F, Rode G, Reilly K, Hadj-Bouziane F, et al. Neuropsychological assessment of a single-case with posterior parietal lesion using behavioural testing and resting state fMRI. OBM Neurobiology. 2021;5(3):20.

[44] Fernandez-Ruiz J, Goltz HC, DeSouza JF, Vilis T, Crawford JD. Human parietal "reach region" primarily encodes intrinsic visual direction, not extrinsic movement direction, in a visual motor dissociation task. Cereb Cortex. 2007;17:2283–2292. doi: 10.1093/cercor/bhl137 17215478

[45] Medina J, Jax SA, Coslett HB. Impairments in action and perception after right intraparietal damage. Cortex. 2020;122:288–299. doi: 10.1016/j.cortex.2019.02.004 30879643

[46] Cheviet A, Pisella L, Pélisson D. The posterior parietal cortex processes visuo-spatial and extra-retinal information for saccadic remapping: A case study. Cortex. 2021;139:134–151. doi: 10.1016/j.cortex.2021.02.026 33862400

[47] Pisella L, Striemer C, Blangero A et al. Perceptual deficits in optic ataxia? In: Book: sensorimotor foundations of higher cognition: attention and performance XXII, Chapter: 3, Publisher, Oxford University Press; 2007. pp. 47–72.

[48] Pisella L, Blangero A, Tilikete C, Biotti D, Rode G, Vighetto A, et al. Attentional disorders. Chapter 16 In: Ochsner Kevin and Kosslyn Stephen (Eds): The Oxford Handbook of Cognitive Neuroscience: Volume 1 Core Topics. Oxford University Press, Oxford, UK; 2013. pp. 319–350.

[49] Pisella L, Vialatte A, Khan AZ, Rossetti Y. Balint syndrome. In; Handbook of Clinical Neurology - Neurology of Vision, Barton J. & Leff A. Eds, Elsevier; 2021. pp. 178:233–255. 10.1016/B978-0-12-821377-3.00011-8 33832679

